# Opium abuse and stroke in Iran: A systematic review and meta-analysis

**DOI:** 10.3389/fneur.2022.855578

**Published:** 2022-09-09

**Authors:** Parham Mardi

**Affiliations:** Non-communicable Diseases Research Center, Alborz University of Medical Sciences, Karaj, Iran

**Keywords:** stroke, cerebrovascular, opium, *Papaver somniferum*, ischemic

## Abstract

**Introduction:**

Opium dependence is a significant health concern in low and middle-income countries, leading to a considerable number of deaths annually. Opium has several detrimental effects on its consumers. Data regarding the impact of opium on stroke are controversial. The objective of this study is to evaluate the association between opium dependence and stroke.

**Methods:**

I conducted a systematic search based on Preferred Reporting Items for Systematic Reviews and Meta-Analyses (PRISMA) guidelines to evaluate the association between opium dependence and stroke. Following the extraction of qualitative findings from included studies, a meta-analysis was performed to assess the pooled estimate of odds ratios (ORs).

**Results:**

Eight and four studies were included in qualitative and quantitative synthesis, respectively. Opium dependence increases the hazard of stroke mortality. Also, opium increases the odds of ischemic stroke by 127% (pooled OR = 2.27, 95% CI: 1.47–3.07).

**Conclusion:**

Opium not only merely increases the odds of being diagnosed with ischemic stroke but also leads to a notable increase in the mortality rate following stroke.

## Introduction

Opium (*Lachryma papaveris*) abuse has led to 1,000's of deaths annually. It is estimated that about 30 million people are addicted to opium or its derivatives (1). Not merely, these people are at increased risk of multiple diseases (2), but also they are at higher hazard of mortality as a result of malignancies, cardiovascular disease, respiratory disease, and infections. Golestan Cohort Study (GCS), a prospective study focusing on the effects of opium conducted in northern Iran, estimated that ever-use of opium results in an 86% higher hazard of all-cause mortality (3). They found that 16.95% of their participants are opium dependent. In a broader view, the World Health Organization estimates that about 2 million Iranians abuse opium, which is three times higher than the global average (4).

GCS also revealed that stroke accounts for 14.0% of premature death (5). The burden imposed by stroke increases as life expectancy rises, especially in low and middle-income countries where incidence or mortality rates have not been altered significantly (6–9).

Some studies justify these epidemiological findings by the surge in well-established risk factors such as hypertension, atrial fibrillation, diabetes, dyslipidemia, aging, and migraine (10–12). Moreover, several studies aimed to search for novel risk factors to explain the residual risk unaccounted by the set of these well-established risk factors (13, 14). With regards to opium dependence's medical and epidemiological features, I designed a systematic review focusing on the association between opium dependence and stroke.

## Methods

This study is conducted based on the preferred reporting items for systematic reviews and meta-analyses (PRISMA) guidelines (15).

### Eligibility criteria

I have used the following eligibility criteria: (I) studies should report the characteristics of patients diagnosed with opium dependence; (II) studies should report characteristics of patients diagnosed with any kind of stroke; (III) studies should report the association between opium with stroke incidence, prevalence, and outcome; and (IV) studies should be observational (namely, cohort, case-control, or cross-sectional studies).

### Information sources

A systematic search was conducted through PubMed, Scopus, and Web of Science (WoS), from inception, until January 12, 2022. I also carried out a manual search using google scholar.

### Search strategy

The search strategy for the systematic search was designed comprising two concepts; stroke and opium. I searched (“stroke” AND “opium”) OR (“cerebrovascular” AND “opium”) OR (“cva” AND “opium”) OR (“apoplexy” AND “opium”) OR (“stroke” AND “papaver”) OR (“cerebrovascular” AND “papaver”) OR (“cva” AND “papaver”) OR (“apoplexy” AND “papaver”). The search strategy algorithm is also shown in Supplementary Table 1.

### Study selection

The study selection was carried out *via* the EndNote reference management software in three phases to manage identified records. Initially, duplicate articles were removed using the endnote software and manually, followed by the screening phase, in which the title and abstract of the studies were evaluated based on the inclusion criteria. Afterward, the screening of the full texts was conducted.

### Data collection process and data items

I filled data extraction sheets containing variables such as author name, year of publication, age, gender, study type, sample size, the definition of exposure and outcome, the measure of association, and study findings.

### Quality assessment

Quality assessment (QA) of included studies was conducted by STrengthening the Reporting of OBservational studies in Epidemiology (STROBE) guideline, comprising 22 sub-items. Each item's ratings are yes (1) or no (0), and the total QA score is the sum of these sub-items. This guideline restricts its recommendations to the three main analytical designs used in observational research (cohort, case-control, and cross-sectional studies) (16).

### Data synthesis

The findings of this study are presented as odds ratios (ORs) and 95% confidence interval (95% CI). In order to perform the meta-analysis, STATA version 14.0 (StataCorp, College Station, TX) software was used. I restrict the meta-analysis when two or more records report the association of opium and stroke. The pooled estimate of ORs and their 95% CI was calculated based on the extracted datasheet. *I*^2^ statistic and chi-square-based Q-test were used for the assessment of heterogeneity. In the current study, the lack of heterogeneity was specified when the *p*-value was more than 0.10. Random models were used to pool the ORs. Publication bias was measured using Begg's test. Publication bias was considered substantial whenever the *p*-value was <0.1.

## Results

### Study selection

**Figure 1:**
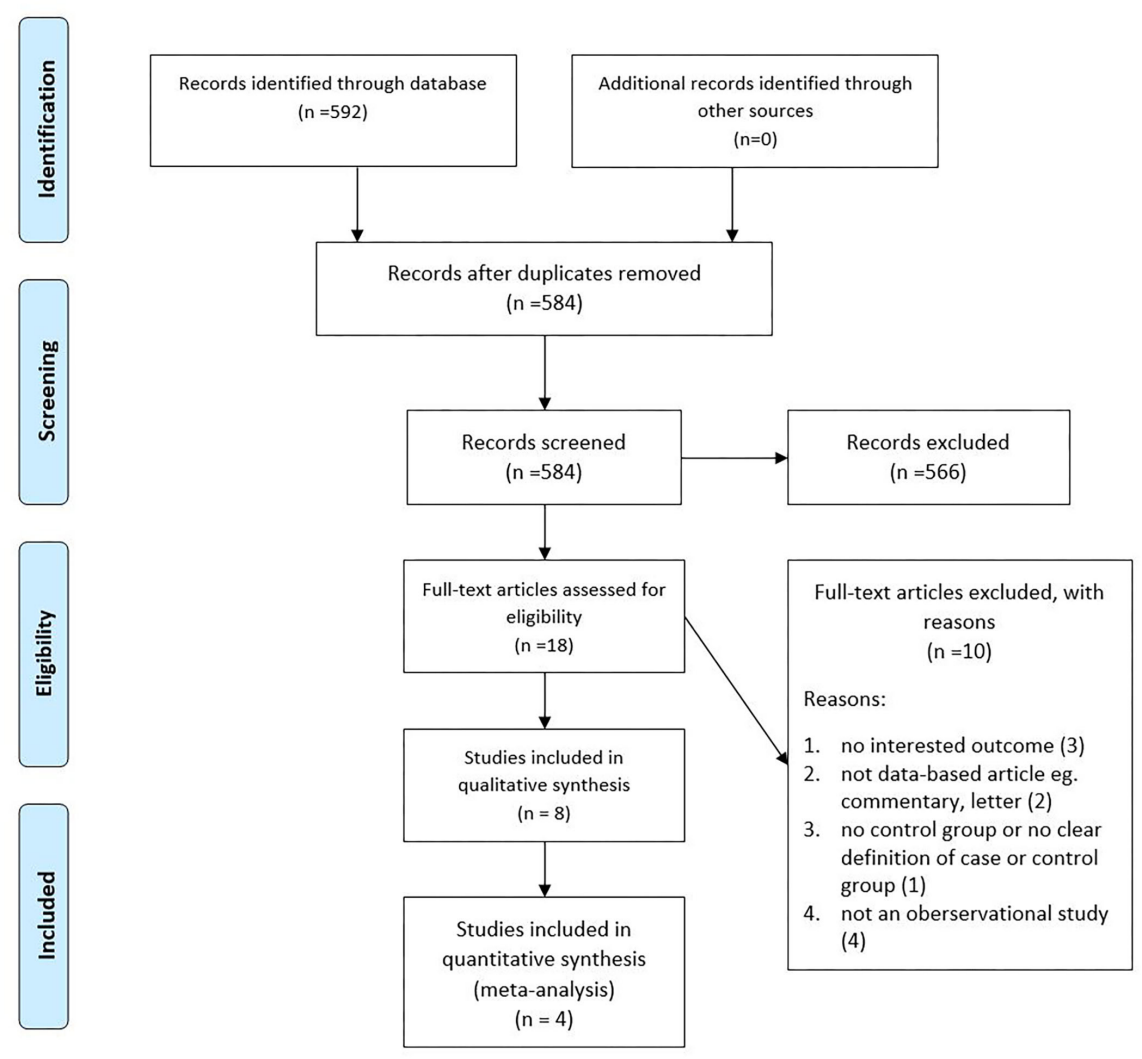
PRISMA flow diagram of included studies.

Our manual and systematic searches yielded 110 studies from PubMed, 88 studies from Scopus, 92 studies from Web of Science, and 302 from Google scholar. After duplicates removal, I evaluated 584 studies for eligibility, followed by the exclusion of 566 records based on their title and abstract. Finally, eight and four studies were included in the qualitative and quantitative syntheses. The detailed flow diagram is shown in Figure 1.

### Study characteristics

Table 1 demonstrates the characteristics of included studies (3, 17–23). Six (75%) studies included in the qualitative analysis were case-control, while the remaining two records were cohort studies. The sample size of included studies ranges from 133 in the Mousavi-Mirzaei et al. study (21) to 50,045 in the Khademi et al. study. The highest percentage of men in a paper was 57.8 in Saberi et al. study. QA of Studies based on STROBE showed that the QA score of included studies ranges from 15 to 20 out of 22.

**Table 1:** Demographic characteristics of participants of included studies.

**References**	**Study type**	**Provenance**	**Sample size**	**Male (%)**	**Age**	**Demographic characteristics**	**Quality assessment score (out of 22)**
Andalibi et al. (17)	Prospective cohort	Iran	595	308 (51.76)	64.6 ± 14.8	• **Study Name:** Mashhad Stroke Incidence Study • **Time period:** 12-month period (2006–2007) • **Study location:** Mashad, an urban area of Iran • **Other features of study population:** 5 points follow-up, including 1 week, 1 month, 3 months, 1 year, and 5 years after their stroke	20
Ebrahimi et al. (18)	Case-control	Iran	956	542 (56.69)	67.19	• **Time period:** 2014 • **Study location:** Alzahra Hospital in Isfahan, Iran • **Other features of study population:** case = patients with confirmed AIS, control = hospitalized patients with no history of AIS and cardiovascular disease, matched for age (±5 years) and sex.	16
Fallahzadeh et al. (19)	Case-control	Iran	9,264	4,276 (46.15)	52.6 ± 9.7	• **Study Name:** The Pars Cohort Study • **Time period:** Fall 2012 to 2014 • **Study location:** Valashahr district, a rural area consisting of Valashahr city and 93 villages located in Fars province in southern Iran. • **Other features of study population:** study population was consist of a variety of ethnicities, mainly Fars and Turk.	17
Hamzehee Moghadam et al. (20)	Case-control	Iran	210	100 (47.6)	66.9	• **Study location:** Shafa hospital, Kerman, Iran • **Other features of study population:** Patients diagnosed with Acute ischemic stroke.	15
Khademi et al. (3)	Prospective cohort	Iran	50,045	21,019 (42.00)	52.1 ± 8.9	• **Study Name:** The Golestan Cohort Study • **Time period:** January 2004 to June 2008 • **Study location:** Gonbad City and 326 villages in Golestan province, north eastern Iran	19
Mousavi-Mirzaei et al. (21)	Case-control	Iran	133	70 (52.63)	71.52	• **Time period:** June 2016 to April 2017 • **Study location:** Vali-Asr Hospital of Birjand, East of Iran. • **Other features of study population:** patients diagnosed with AIS.	17
Rezvani and Ghandehari (22)	Case-control	Iran	558	251 (44.98)	56.2	• **Time period:** 2010 • **Study location:** Cardiology and Neurology clinics of Birjand, Province of Southern Khorasan, Iran.	18
Saberi et al. (23)	Case-control	Iran	166	96 (57.8)	68.2	• **Time period:** April 2013 to March 2014 • **Study location:** two academic hospitals associated with Guilan University of Medical Science in north of Iran.	15

AIS, Acute ischemic stroke was defined as a sudden focal neurologic deficit of presumed arterial origin lasting ≥24 h with or without corresponding ischemic lesion on brain imaging.

Age is described as mean or mean ± standard deviation.

Gender is described as number of males (percent).

#### Qualitative findings

Six included studies evaluated the relationship between opium consumption and stroke (18–23). The outcome of four papers was the diagnosis of ischemic stroke (18, 20, 22, 23), while in two other, indices regarding the formation of ischemic plaque (21) and diagnosis of any kind of stroke (19) were the outcome. Four (18, 19, 21, 22) out of these six studies revealed at least a significant measure concerning the association between stroke and opium use.

Moreover, a single paper was driven from GCS revealing that opium use increases the hazard of mortality due to stroke (3). Similarly, Andalibi et al.'s study showed that although opium use increases the hazard of death following stroke, it does not significantly alter the hazard of disability following stroke (17). The qualitative synthesis of included studies is shown in Table 2.

**Table 2:** Qualitative findings of included studies.

**References**	**Study type**	**Exposure**	**Outcome**	**Measure of association**	**Findings**	
Andalibi et al. (17)	Prospective cohort	Opium use (burned and	Mortality following	Adjusted hazard	7 days	1.36 (0.70–2.65)
		the sap)	any type of stroke	ratio (95% CI)	1 month	1.51 (0.91–2.51)
			Disability following		3 months	1.58 (1.01–2.49)
			stroke (mRS^*^)		1 year	1.78 (1.20–2.65)
					5 years	1.75 (1.28–2.40)
					3 months	0.63 (0.23–1.67)
					1 year	1.19 (0.42–3.37)
					5 years	0.54 (0.09–3.22)
Ebrahimi et al. (18)	Case-control	Opium addiction (based on medical records and interview of the patient)	Ischemic stroke	Adjusted odds ratio (95% CI)	Not adjusted for tobacco use Adjusted for tobacco use	3.95 (2.02–7.69) 2.36 (1.16–4.85)
Fallahzadeh et al. (19)	Case-control	Self-report opium use	Stroke (all types)	Unadjusted odds ratio (95% CI)		1.60 (1.01–2.55)
Hamzehee-Moghadam	Case-control	Constant use of opium for at least 1 year	Ischemic stroke	Logistic regression (95% CI)	β (SE)	0.861(0.316)
et al. (20)				Adjusted odds ratio (95% CI)	Adjusted odds ratio	2.36 (1.26–3.61)
Khademi et al. (3)	Cohort	Opium use (self-report)	Death as a result of	Adjusted hazard	Male	1.49 (1.07–2.08)
			any kind of stroke	ratio (95% CI)	Female	1.97 (1.30–2.97)
					Total	1.68 (1.29–2.18)
Mousavi-Mirzaei	Case-control	Opium dependence^**^	Atherosclerotic	Adjusted odds ratio	Number of plaques	1.42 (1.11–1.81)
et al. (21)			plaques	(95% CI)	Vascular stenosis	1.01 (0.98–1.03)
					Calcified plaque	1.37 (0.95–1.97)
					Internal media thickness	2.48 (2.27–10.94)
Rezvani et al. (22)	Case-control	Opium addiction^**^	Ischemic stroke	Adjusted odds ratio (95% CI)	Oral opium addiction	0.21 (0.08–0.56)
					Inhaled opium addiction	1.76 (0.76–4.08)
Saberi et al. (23)	Case-control	Opium addiction^**^	Ischemic stroke	Logistic regression	β (SE)	0.67 (0.40)
				Adjusted odds ratio (95% CI)	Adjusted odds ratio	1.94 (0.89–4.36)

* The Modified Rankin Scale (mRS) is used to measure the degree of disability in patients who have had a stroke.

** Opium addiction is diagnosed when three of seven physiological (e.g., tolerance and withdrawal); behavioral (e.g., taking the drug in larger amounts than intended), and cognitive (e.g., persistent desire to cut down) symptoms are met within the previous 12 months.

CI, confidence interval.

#### Quantitative findings

**Figure 2:**
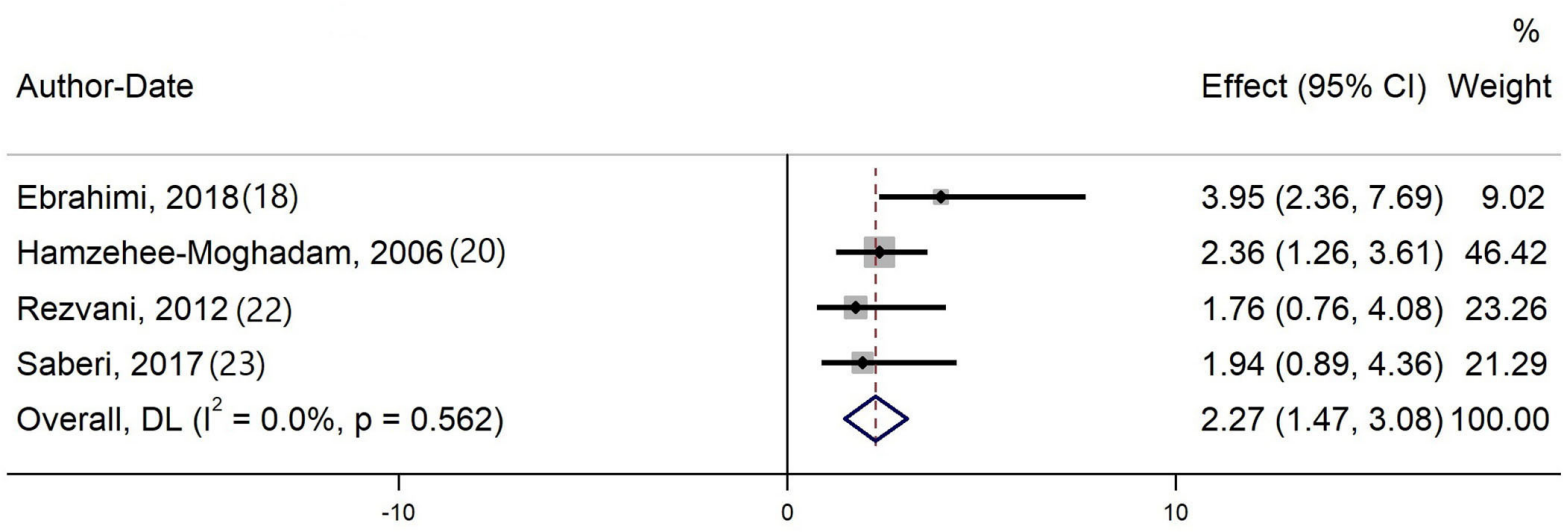
Forest plot of the meta-analysis.

Four studies evaluating the effect of opium on ischemic stroke were included in the meta-analysis (18, 20, 22, 23). The quantitative synthesis proved that opium increases the odds of ischemic stroke by 127% (pooled OR = 2.27, 95% CI: 1.47–3.07). No heterogeneity was evident in current meta-analysis (Cochran's *Q* = 2.05, degree of freedom = 3, *p*-value = 0.562, *I*^2^ = 0.0%). Also, Begg's test did not show a significant publication bias (*p*-value = 0.192). Forest plot of the meta-analysis is shown in Figure 2.

### Discussion

This study indicated that not only opium dependence almost doubles the odds of being diagnosed with a stroke but also it leads to a higher hazard of mortality due to stroke. This is the first systematic review and meta-analysis that proposes opium dependence as a notable risk factor for stroke to the best of my knowledge. It should be clarified that this study and included records are all focused on the cerebrovascular effects of opium abuse, rather than the effects of prescribed opium, as a component of a medical treatment plan.

Our meta-analysis on multi-variable adjusted case-control studies revealed that opium consumption independently increases the odds of ischemic stroke. Moadabi et al. proposed hypothetical pathophysiology underlying the impact of opium on cerebral hemodynamic parameters. They performed transcranial Doppler (TCD) ultrasonography, which revealed that patients with opium dependence are at higher odds of abnormal pulsatility index (OR = 9.615) and mean flow velocity (OR = 3.246). In other words, they indicated that opium affects both small and large vessels, which leads to a higher risk of ischemic stroke (24). Likewise, Mousavi-Mirzaei et al.'s study revealed that opium consumption leads to increased atherosclerotic plaques and internal media thickness, a well-established component of the pathogenesis of ischemic stroke (21). These findings are similar to studies showing an increased risk of myocardial infarction and coronary revascularization, among opium users (25). Opium consumption is a significant risk factor for coronary artery disease, as it increases the progression of atherosclerosis, and leads to an increase in low-density lipoprotein cholesterol (LDL-C) level and a decrease in high-density lipoprotein cholesterol through opium-induced activation of hepatic pathways (26–28).

Moreover, the Asiabanha et al. study demonstrated that opium imposes brain cell damage by inducing apoptosis, which may prone the brain to cerebrovascular incidents (29). Opium addiction also affects endothelial function, leading to a decline in nitrous oxide level (30), a molecule that improves brain blood flow and neuronal survival in stroke (31).

Apart from previously mentioned pathways, opium addiction is a well-established risk factor for diabetes, hypertension, and hyperlipidemia (32), which can potentially elevate stroke hazards (33, 34). Studies, included in the current meta-analysis, were adjusted for potential confounders such as dyslipidemia, diabetes, and hypertension. Nevertheless, it should be noted that previous studies revealed that opium consumption worsens glucose metabolism in patients both with diabetes and non-diabetes (35, 36), and it results in a significant disturbance in lipid profile, which in turn may provoke stroke (28). To sum up, opium not only directly affects brain cells but also leads to unfavorable changes in the incidence of metabolic risk factors.

Another modifiable risk factor for stroke is tobacco smoking. Comparing the effects of tobacco smoking and opium as habitual risk factors can help understand the harmful but modifiable effects of opium. OR calculated for the effect of opium on stroke in this study was higher or similar compared to ORs calculated for tobacco smoking (37–41). Pan et al.'s meta-analysis revealed that current smokers are at higher odds of stroke (1.92, 95% CI: 1.49–2.48), while the association of being a former smoker and stroke was not significant (1.30, 95% CI: 0.93–1.81) (38). Likewise, Wannamethee et al. study showed that quitting smoking results in notable and rapid benefits in reducing the risk of stroke (42). Interestingly, smoking cessation even after stroke enhances the outcome (43). Beneficial effects of opium cessation are also evident in previous studies (3, 44), although there is no scientific evidence regarding the impact of opium cessation and a decline in stroke risk.

Smoking is generally more prevalent in men (45). Similarly, the prevalence of opium addiction is notably higher in men than women (3). As the number of studies included in my meta-analysis was few and no heterogeneity was evident, I could not conduct a meta-regression analysis to evaluate the effect of gender. However, it is worth noting that experimental studies showed that addicted men are at higher risk of ischemic stroke as men brain cells are more susceptible to opium than women (29).

Our review shows that opium predisposes patients to stroke and increases the hazard of mortality following any type of stroke. In other words, Khademi et al.'s study demonstrated that opium consumption leads to strongly increased risks of death due to a stroke. That is to say, in GCS, opium was the cause of mortality in 0.50% of non-users compared to 1.15% of participants who consume opium. These findings remained significant after adjustment for potential confounders (3).

Comparably, Andalibi et al.'s study illustrated that using opium increases mortality risk following stroke. More specifically, multi-regression models proved that patients who consume opium are at higher hazard of 3-months (HR = 1.58, 95% CI: 1.01–2.49), 1-year (HR = 1.78, 95% CI: 1.20–2.65), and 5-years (HR = 1.75, 95% CI: 1.28–2.40) mortality. Conversely, patients' disability was not significantly different in user and non-user groups. Participants' disability was measured using an objective index (Modified Rankin Scale), which can be influenced by the analgesic and euphoric effects of opium (17).

Given the remarkable effects of opium on stroke, addiction prevention programs should be seriously pursued in countries where opium dependence is common, namely, central Asian countries and Iran (46). Although I did not add a filter for the provenance of the study, all included studies were from Iran in the current review. Even though the single geographical provenance of included studies makes the generalization of my findings challenging, it can assist other low and middle-income countries, similar to Iran, in adopting better policies.

### Strengths and limitations

This study had several strengths. Most importantly, this is the first systematic review and meta-analysis that evaluates the effects of opium on stroke. This study evaluates the effect of opium on stroke incidence and mortality, and it also reviewed papers that evaluated the outcome of patients dependent on opium following stroke. This study also had several limitations; first, the number of studies included in the quantitative analysis was limited, resulting in a bias. Second, all included studies were from a single country (Iran). Third, studies included in the meta-analysis were all case-control studies. Conduction of larger cohorts is suggested to assess the effect of opium more precisely as a risk factor. Fourth, due to the absence of randomization, *p*-values (even underlying the confidence intervals) must be considered as a possible approximation to reality.

## Conclusion

This study shows that opium consumption significantly increases the odds of being diagnosed with stroke in the general population. Moreover, this study's findings indicate that not only might there be a significant relationship between opium and mortality as a result of stroke but opium may also increase the odds of death after stroke.

## Data availability statement

The raw data supporting the conclusions of this article will be made available by the authors, without undue reservation.

## Author contributions

PM conceived the idea for the topic, performed the review, conducted quantitative synthesis, and drafted the manuscript.

## Conflict of interest

The author declares that the research was conducted in the absence of any commercial or financial relationships that could be construed as a potential conflict of interest.

## Publisher's note

All claims expressed in this article are solely those of the authors and do not necessarily represent those of their affiliated organizations, or those of the publisher, the editors and the reviewers. Any product that may be evaluated in this article, or claim that may be made by its manufacturer, is not guaranteed or endorsed by the publisher.

## References

[1] Drugs U Crime M. World Drug Report 2010. New York, NY: United Nations Publications (2010).

[2] Sheikh M Kamangar F Malekzadeh R. Fifty years of research and one conclusion: opium causes cancer. Arch Iran Med. (2020) 23:757–60. 10.34172/aim.2020.95 33220692

[3] Khademi H Malekzadeh R Pourshams A Jafari E Salahi R Semnani S . Opium use and mortality in Golestan Cohort Study: prospective cohort study of 50,000 adults in Iran. Br Med J. (2012) 344:e2502. 10.1136/bmj.e2502 22511302 PMC3328545

[4] Eskandarieh S Jafari F Yazdani S Hazrati N Saberi-Zafarghandi MB. Compulsory maintenance treatment program amongst Iranian injection drug users and its side effects. Int J High Risk Behav Addict. (2014) 3:21765. 10.5812/ijhrba.21765 25741482 PMC4331656

[5] Nalini M Oranuba E Poustchi H Sepanlou SG Pourshams A Khoshnia M . Causes of premature death and their associated risk factors in the Golestan Cohort Study, Iran. BMJ Open. (2018) 8:e021479. 10.1136/bmjopen-2018-021479 30021753 PMC6059279

[6] Katan M Luft A. Global burden of stroke. Semin Neurol. (2018) 38:208–11. 10.1055/s-0038-1649503 29791947

[7] Mensah GA Norrving B Feigin VL. The global burden of stroke. Neuroepidemiology. (2015) 45:143–5. 10.1159/000441082 26505979 PMC4631389

[8] Collaborators GLRoS. Global, regional, and country-specific lifetime risks of stroke, 1990 and 2016. N Engl J Med. (2018) 379:2429–37. 10.1056/NEJMoa1804492 30575491 PMC6247346

[9] Feigin VL Krishnamurthi RV Parmar P Norrving B Mensah GA Bennett DA . Update on the global burden of ischemic and hemorrhagic stroke in 1990-2013: the GBD 2013 study. Neuroepidemiology. (2015) 45:161–76. 10.1159/000441085 26505981 PMC4633282

[10] van Alebeek ME Arntz RM Ekker MS Synhaeve NE Maaijwee NA Schoonderwaldt H . Risk factors and mechanisms of stroke in young adults: the FUTURE study. J Cereb Blood Flow Metab. (2018) 38:1631–41. 10.1177/0271678X17707138 28534705 PMC6120122

[11] Bitew ZW Alemu A Ayele EG Tenaw Z Alebel A Worku T. Metabolic syndrome among children and adolescents in low and middle income countries: a systematic review and meta-analysis. Diabetol Metab Syndr. (2020) 12:1–23. 10.1186/s13098-020-00601-8 33117455 PMC7590497

[12] Borch-Johnsen K. The metabolic syndrome in a global perspective. The public health impact–secondary publication. Dan Med Bull. (2007) 54:157–9. 17521535

[13] Freedman B Martinez C Katholing A Rietbrock S. Residual risk of stroke and death in anticoagulant-treated patients with atrial fibrillation. J Am Med Assoc Cardiol. (2016) 1:366–8. 10.1001/jamacardio.2016.0393 27438123

[14] Pan Y Li Z Li J Jin A Lin J Jing J . Residual risk and its risk factors for ischemic stroke with adherence to guideline-based secondary stroke prevention. J Stroke. (2021) 23:51. 10.5853/jos.2020.03391 33600702 PMC7900402

[15] Tricco AC Lillie E Zarin W O'Brien KK Colquhoun H Levac D . PRISMA extension for scoping reviews (PRISMA-ScR): checklist and explanation. Ann Intern Med. (2018) 169:467–73. 10.7326/M18-0850 30178033

[16] Vandenbroucke JP Von Elm E Altman DG Gøtzsche PC Mulrow CD Pocock SJ . Strengthening the Reporting of Observational Studies in Epidemiology (STROBE): explanation and elaboration. PLoS Med. (2007) 4:e297. 10.1371/journal.pmed.0040297 17941715 PMC2020496

[17] Andalibi MSS Rezaei Ardani A Amiri A Morovatdar N Talebi A Azarpazhooh MR . The association between substance use disorders and long-term outcome of stroke: results from a population-based study of stroke among 450,229 urban citizens. Neuroepidemiology. (2021) 55:171–9. 10.1159/000514401 33975326

[18] Ebrahimi H Javanmard SH Asgary S Dehghani L Amiri M Saadatnia M. Opium addiction and Ischemic stroke in Isfahan, Iran: a case-control study. Eur Neurol. (2018) 79:82–5. 10.1159/000485098 29275418

[19] Fallahzadeh MA Salehi A Naghshvarian M Fallahzadeh MH Poustchi H Sepanlou SG . Epidemiologic study of opium use in pars cohort study: a study of 9,000 adults in a rural Southern Area of Iran. Arch Iran Med. (2017) 20:205–10. 28412823

[20] Hamzehee Moghadam A Mousavi A. The relationship between opium dependency and stroke. J Kerman Univ Medical Sci. (2006) 13:203–8.

[21] Mousavi-Mirzaei SM Talebi A Amirabadizadeh A Nakhaee S Azarkar G Mehrpour O. Increasing the risk of stroke by opium addiction. J Stroke Cerebrovasc Dis. (2019) 28:1930–5. 10.1016/j.jstrokecerebrovasdis.2019.03.044 31000450

[22] Rezvani MR Ghandehari K. Is opium addiction a risk factor for ischemic heart disease and ischemic stroke? J Res Medical Sci. (2012) 17:958–61. 23825997 PMC3698656

[23] Saberi A Ghayeghran AR Janeshin S Biazar G Kazemnezhad E. Opium consumption prevalence among patients with ischemic stroke compared with healthy individuals in Iran. Int J High Risk Behav Addict. (2017) 6:e27264. 10.5812/ijhrba.27264

[24] Moadabi Y Saberi A Hoseini S Karimi A Yousefzadeh-Chabok S. Cerebral hemodynamic abnormalities of patients with ischemic stroke who are opium addicted: a study by transcranial doppler ultrasonography. Iran J Neurol. (2019) 18:76–81. 31565204 PMC6755506

[25] Chow SL Sasson C Benjamin IJ Califf RM Compton WM Oliva EM . Opioid use and its relationship to cardiovascular disease and brain health: a presidential advisory from the American Heart Association. Circulation. (2021) 144:e218–32. 10.1161/CIR.0000000000001007 34407637

[26] Bryant HU Kuta CC Story JA Yim GK. Stress- and morphine-induced elevations of plasma and tissue cholesterol in mice: reversal by naltrexone. Biochem Pharmacol. (1988) 37:3777–80. 10.1016/0006-2952(88)90415-7 3178891

[27] Maron DJ. The epidemiology of low levels of high-density lipoprotein cholesterol in patients with and without coronary artery disease. Am J Cardiol. (2000) 86:11l−4l. 10.1016/S0002-9149(00)01462-4 11374848

[28] Mohammadi A Darabi M Nasry M Saabet-Jahromi MJ Malek-Pour-Afshar R Sheibani H. Effect of opium addiction on lipid profile and atherosclerosis formation in hypercholesterolemic rabbits. Exp Toxicol Pathol. (2009) 61:145–9. 10.1016/j.etp.2008.08.001 18838257

[29] Asiabanha M Asadikaram G Rahnema A Mahmoodi M Hasanshahi G Hashemi M . Chronic opium treatment can differentially induce brain and liver cells apoptosis in diabetic and non-diabetic male and female rats. Kjpp. (2011) 15:327–32. 10.4196/kjpp.2011.15.6.327 22359469 PMC3282219

[30] Ghanei AM Saadatnia M Javanmard SH. Effects of opium addiction on vascular endothelium. J Isfahan Medical School. (2013) 2084–90. Available online at: http://jims.mui.ac.ir/index.php/jims/about/article_13954.html?lang=en

[31] Terpolilli NA Kim SW Thal SC Kataoka H Zeisig V Nitzsche B . Inhalation of nitric oxide prevents ischemic brain damage in experimental stroke by selective dilatation of collateral arterioles. Circul Res. (2012) 110:727–38. 10.1161/CIRCRESAHA.111.253419 22207711

[32] Najafipour H Beik A. The impact of opium consumption on blood glucose, serum lipids and blood pressure, and related mechanisms. Front Physiol. (2016) 7:436. 10.3389/fphys.2016.00436 27790151 PMC5061814

[33] Hong K-S Bang OY Kang DW Yu KH Bae HJ Lee JS . Stroke statistics in Korea: part I. Epidemiology and risk factors: a report from the korean stroke society and clinical research center for stroke. J Stroke. (2013) 15:2. 10.5853/jos.2013.15.1.2 24324935 PMC3779679

[34] Wang J Wen X Li W Li X Wang Y Lu W. Risk factors for stroke in the Chinese population: a systematic review and meta-analysis. J Stroke Cerebrovasc Dis. (2017) 26:509–17. 10.1016/j.jstrokecerebrovasdis.2016.12.002 28041900

[35] Asgary S Sarrafzadegan N Naderi GA Rozbehani R. Effect of opium addiction on new and traditional cardiovascular risk factors: do duration of addiction and route of administration matter? Lipids Health Dis. (2008) 7:1–5. 10.1186/1476-511X-7-42 18980684 PMC2588593

[36] Karam GA Reisi M Kaseb AA Khaksari M Mohammadi A Mahmoodi M. Effects of opium addiction on some serum factors in addicts with non-insulin-dependent diabetes mellitus. Addict Biol. (2004) 9:53–8. 10.1080/13556210410001674095 15203439

[37] Hankey GJ. Smoking and risk of stroke. J Cardiovasc Risk. (1999) 6:207–11. 10.1177/204748739900600403 10501270

[38] Pan B Jin X Jun L Qiu S Zheng Q Pan M. The relationship between smoking and stroke: a meta-analysis. Medicine. (2019) 98L14872. 10.1097/MD.0000000000014872 30896633 PMC6708836

[39] Shah RS Cole JW. Smoking and stroke: the more you smoke the more you stroke. Expert Rev Cardiovasc Ther. (2010) 8:917–32. 10.1586/erc.10.56 20602553 PMC2928253

[40] Shinton R Beevers G. Meta-analysis of relation between cigarette smoking and stroke. Br Medical J. (1989) 298:789–94. 10.1136/bmj.298.6676.789 2496858 PMC1836102

[41] Wolf PA D'Agostino RB Kannel WB Bonita R Belanger AJ. Cigarette smoking as a risk factor for stroke: the Framingham Study. J Am Med Assoc. (1988) 259:1025–9. 10.1001/jama.259.7.1025 3339799

[42] Wannamethee SG Shaper AG Whincup PH Walker M. Smoking cessation and the risk of stroke in middle-aged men. J Am Med Assoc. (1995) 274:155–60. 10.1001/jama.274.2.155 7596004

[43] Epstein KA Viscoli CM Spence JD Young LH Inzucchi SE Gorman M . Smoking cessation and outcome after ischemic stroke or TIA. Neurology. (2017) 89:1723–9. 10.1212/WNL.0000000000004524 28887378 PMC5644463

[44] Golshiri A Mokhtaree MR Shabani Z Tabatabaee ST Rahnama A Moradi M . Effects of opium smoking cessation on the nasopharyngeal microbial flora. Addict Health. (2009) 1:1. 24494075 PMC3905491

[45] Bauer T Göhlmann S Sinning M. Gender differences in smoking behavior. Health Econ. (2007) 16:895–909. 10.1002/hec.1259 17619229

[46] Ray R Kattimani S Sharma H. Opium Abuse and Its Management: Global Scenario. New Delhi: World Health Organization Department of Mental Health and Substance Abuse Management of Substance Abuse. National Drug Dependence Treatment Centre All India Institute of Medical Sciences (2006). p. 1–13.

