# Supplementary material for: Opium abuse and stroke in Iran: A systematic review and meta-analysis
Source: Front Neurol. 2022 Sep 9;13:855578. doi: 10.3389/fneur.2022.855578 (PMC9524459; doi:10.3389/fneur.2022.855578)
Supplement: Supplementary file 1 [file Table_1.docx]

**Supplementary table**

| 1. | “Stroke” OR “cerebrovascular” OR “cva” OR “apoplexy” |
| --- | --- |
| 2. | “opium” OR “papaver” |
| 3. | #1 AND #2 |
| Filters | Any language; Publication date (from inception, until January 12th, 2022); |
